# Involvement of the Wbp pathway in the biosynthesis of *Porphyromonas gingivalis* lipopolysaccharide with anionic polysaccharide

**DOI:** 10.1038/srep05056

**Published:** 2014-05-23

**Authors:** Mikio Shoji, Keiko Sato, Hideharu Yukitake, Mariko Naito, Koji Nakayama

**Affiliations:** 1 Division of Microbiology and Oral Infection, Department of Molecular Microbiology and Immunology, Nagasaki University Graduate School of Biomedical Sciences, Nagasaki, 852-8588, Japan

## Abstract

The periodontal pathogen *Porphyromonas gingivalis* has two different lipopolysaccharide (LPS) molecules, O-LPS and A-LPS. We have recently shown that *P. gingivalis* strain HG66 lacks A-LPS. Here, we found that introduction of a wild-type *wbpB* gene into strain HG66 restored formation of A-LPS. Sequencing of the *wbpB* gene from strain HG66 revealed the presence of a nonsense mutation in the gene. The *wbpB* gene product is a member of the Wbp pathway, which plays a role in the synthesis of UDP-ManNAc(3NAc)A in *Pseudomonas aeruginosa*; UDP-ManNAc(3NAc)A is sequentially synthesized by the WbpA, WbpB, WbpE, WbpD and WbpI proteins. We then determined the effect of the PGN_0002 gene, a *wbpD* homolog, on the biosynthesis of A-LPS. A PGN_0002-deficient mutant demonstrated an A-LPS biosynthesis deficiency. Taken together with previous studies, the present results suggest that the final product synthesized by the Wbp pathway is one of the sugar substrates necessary for the biosynthesis of A-LPS.

The periodontal pathogen *Porphyromonas gingivalis* is a Gram-negative obligate anaerobe. As *P. gingivalis* cannot utilize saccharides, the bacterium expresses many proteases on the cell surface to utilize peptides as carbon and nitrogen sources. Specifically, cysteine proteases such as Arg-gingipain (Rgp) and Lys-gingipain (Kgp) are considered important *P. gingivalis* virulence factors^1^.

*P. gingivalis* displays black pigmentation on blood agar plates. The black pigmentation is the result of storage of the μ-oxo-dimeric form of heme (iron protoporphyrin IX) on the cell surface^2^. Kgp can degrade the hemoglobin protein, which holds heme molecules, and it has been shown that the *kgp* mutant possesses a pigment-less phenotype^3^. Previous studies using transposon mutagenesis have revealed that *porR*^4^ and *porT*^5^ are involved in the pigmentation phenotype in *P. gingivalis*. The *porR-*type mutation demonstrates significantly reduced gingipain activities on the cell surface but retains these activities in the culture supernatant, suggesting that the PorR*-*type system plays a role in anchoring the gingipains on the cell surface. Conversely, the *porT-*type mutation results in no gingipain activities on the cell surface or in the culture supernatant, suggesting that the PorT*-*type system is involved in the secretion of gingipains onto the cell surface. In *P. gingivalis*, studies have shown that the PorT*-*type system is composed of 11 proteins, named the Por secretion system (PorSS)/type IX secretion system (T9SS); this system is widely distributed in the *Bacteroidetes* phylum except for *Bacteroides fragilis* and *Bacteroides thetaiotaomicron*^6^,^7^,^8^.

*P. gingivalis* has two different lipopolysaccharide (LPS) molecules, O-LPS and A-LPS. O-LPS has the conventional O-antigen of *P. gingivalis* strain W50 consists of a tetrasaccharide repeat unit composed of -3)- α-_D_-Gal*p*-(1-6)-α-_D_-Glc*p*-(1-4)- α-_L_-Rha*p*-(1-3)-β-_D_-GalNAc*p*-(1-9. A-LPS has a different O-antigen from *P. gingivalis* strain W50 and consists of an anionic polysaccharide (APS) repeat unit^10^,^11^. Curtis et al.^12^ obtained a monoclonal antibody (mAb 1B5) that was originally raised against the catalytic domain of the RgpA protease and was later found to cross-react with A-LPS by recognizing a phosphorylated branched mannan in the APS repeat unit^10^. Recently, we have shown that another monoclonal antibody (mAb TDC-5-2-1) recognizes the O-antigen of O-LPS present in almost all wild-type cells; however, the glycan epitope recognized by this monoclonal antibody has not been identified^13^.

The *porR* mutant has been shown to be immunoreactive to mAb TDC-5-2-1, but not to mAb 1B5, indicating that the *porR* mutant possesses O-LPS, but lacks A-LPS^13^. We have previously reported that the *porR* gene encodes a putative transaminase^4^. However, the exact substrate of the PorR protein has not been identified. As A-LPS is assumed to be a *P. gingivalis* virulence factor, genes involved in A-LPS biosynthesis have been recently identified. To date, *vimA*, *vimE*, *vimF*^14^,^15^, *wbpB*^16^, *waaL*^11^ *ugdA*, *rfa*^17^, *wzy*^9^, *gtfB*^18^, PGN_0242, PGN_0663^19^ *wbaP*, *wzx*, *wzzP*^13^ and *porR* have been demonstrated to be involved in A-LPS biosynthesis. The PorR, WbpB and UgdA proteins are predicted to participate in the initial synthesis of structural sugar(s) in the APS. The Rfa protein is involved in the synthesis of the core oligosaccharide of LPS molecules. WbaP (initial phosphoryl glycosyl transferase onto undecaprenyl monophosphate), Wzx (O-antigen flippase), Wzy (O-antigen polymerase), WzzP (O-antigen chain length regulator) and WaaL (O-antigen ligase) are involved in the biosynthesis of both O-antigen and A-antigen. The VimA, VimE and VimF proteins are acetyltransferase, hypothetical protein and galactosyltransferase proteins, respectively^20^,^21^,^22^.

*P. gingivalis* has approximately 30 proteins (called CTD proteins) that contain a conserved C-terminal domain^23^. Some of these proteins are secreted onto the cell surface via the PorSS/T9SS system and are then bound to A-LPS or secreted in the culture supernatant^6^,^7^,^19^,^24^. RgpB and HBP35 are used as model proteins to analyze the PorSS/T9SS secretion system in *P. gingivalis* because these proteins exhibit diffuse bands on a gel, which is indicative of the A-LPS bound form^19^,^25^,^26^.

Whereas wild-type *P. gingivalis* strains possess membrane associated gingipain activities on the cell surface, *P. gingivalis* strain HG66 has significantly reduced gingipain activities on the cell surface but still retains the activities in the culture supernatant^27^. Some CTD proteins, such as RgpB^28^, peptidyl arginine deaminase^29^, periodontain^30^ and CPG70^31^, have been purified from the culture supernatant of strain HG66. We have recently shown that the latter strain possesses O-LPS, but lacks A-LPS, similar to the *porR* mutant^13^. One aim of this study was to determine the reason for the A-LPS deficiency in strain HG66.

To investigate the lack of A-LPS in HG66, we focused on the relationships between the *ugdA*, *wbpB* and *porR* genes in A-LPS biosynthesis. WbpB belongs to the Wbp pathway, which participates in the synthesis of UDP-2,3-diacetamido-2,3-dideoxy-_D_-mannuronic acid [UDP- ManNAc(3NAc)A], a precursor of the ManNAc(3NAc)A residue in the B-band O-antigen of *Pseudomonas aeruginosa*. For UDP-ManNAc(3NAc)A synthesis, WbpA, WbpB, WbpE, WbpD and WbpI react sequentially on the precursor UDP-*N*-acetyl-glucosamine (UDP-GlcNAc) molecule^32^,^33^,^34^. The UgdA and PorR proteins have high similarity to the WbpA and WbpE proteins, respectively. Therefore, we hypothesized that *P. gingivalis* may have a similar Wbp pathway. Genomic analysis revealed that *ugdA* (PGN_0613) and PGN_1243 were *wbpA* homologs, *wbpB* (PGN_0168) was a *wbpB* homolog, *porR* (PGN_1236) was a *wbpE* homolog, and PGN_0002 was a *wbpD* homolog; no *wbpI* homologs were found.

Our study revealed that the A-LPS deficiency of strain HG66 was the result of a nonsense mutation in the *wbpB* gene, and likewise, Wbp pathway gene mutants were A-LPS deficient.

## Results

### Identification of the gene mutation responsible for A-LPS deficiency in strain HG66

**Figure 1:**
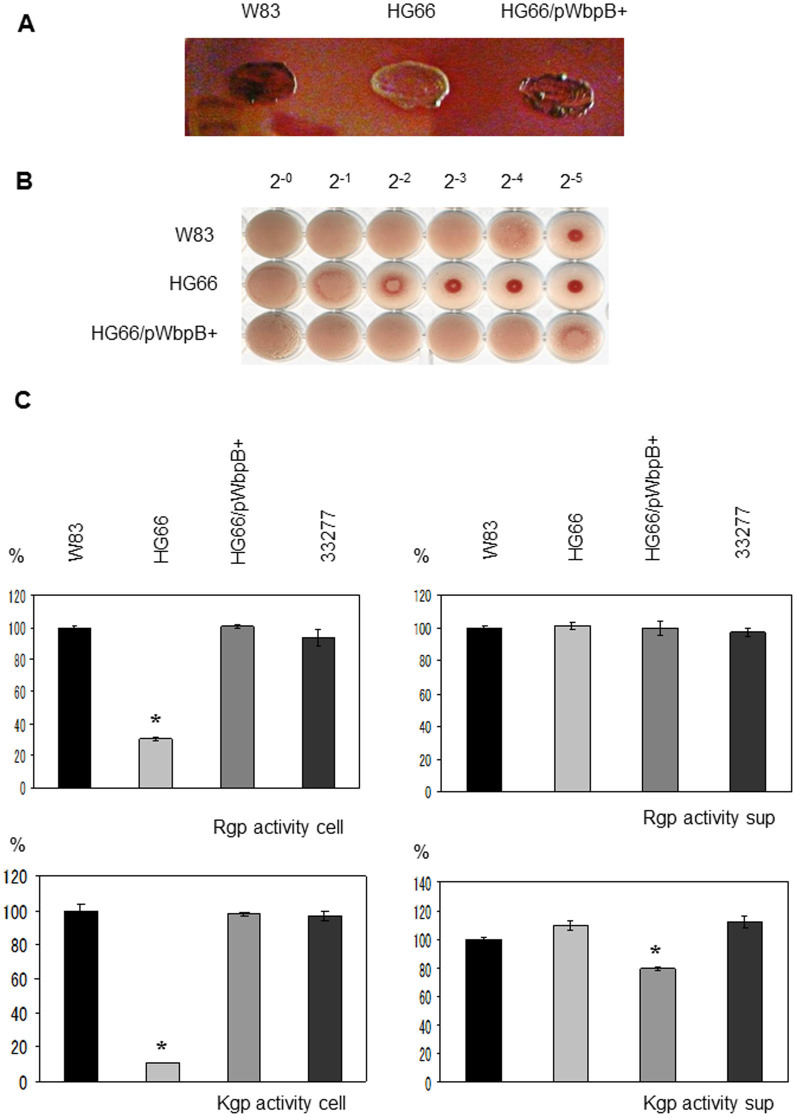
Pigmentation and hemagglutination and gingipain activities of *Porphyromonas gingivalis*. (A) Colony pigmentation of *Porphyromonas gingivalis* cells on blood agar plates. (B) The hemagglutination activities of *P. gingivalis* strains were measured. (C) The Kgp and Rgp activities of the cell lysates (cell) and vesicle-containing culture supernatants (sup) of W83, HG66, HG66/WbpB+ and ATCC 33277 were measured. The mean of each protease activity of W83 was regarded as 100%. Asterisks indicate significant differences in enzymatic activity between W83 and various strains (*P* < 0.01). The bars are expressed as the means ± standard deviation for triplicate samples from one of two independent experiments.

**Figure 2:**
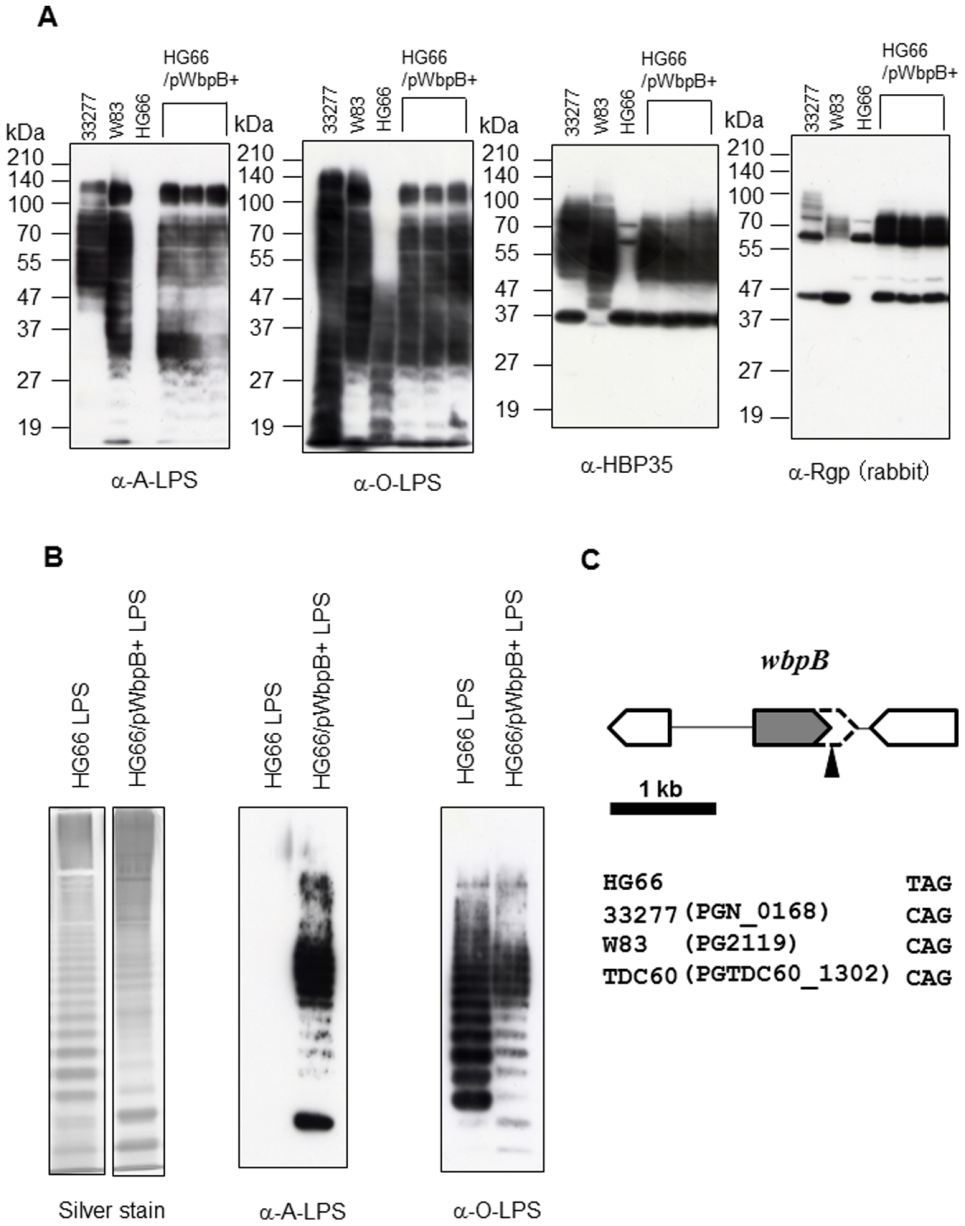
Immunoblot and LPS analyses of *Porphyromonas gingivalis* strain HG66. (A) Immunoblot analyses of cell lysates from various *P. gingivalis* strains were performed with anti-A-LPS (mAb 1B5), anti-O-LPS (mAb TDC-5-2-1), anti-HBP35 or anti-Rgp antibodies. Three sets of HG66/WbpB+ strains were obtained from each clone. (B) The LPS fraction from *P. gingivalis* HG66 or HG66/WbpB+ strain was stained by silver staining. The cropped silver stained gels were run under a same SDS-PAGE gel. Immunoblot analyses were performed with anti-A-LPS (mAb 1B5) or anti-O-LPS (mAb TDC-5-2-1) antibodies. (C) Physical map of the area around the *wbpB* gene in *P. gingivalis*. The arrowhead indicates the nonsense mutation in HG66.

**Figure 3:**
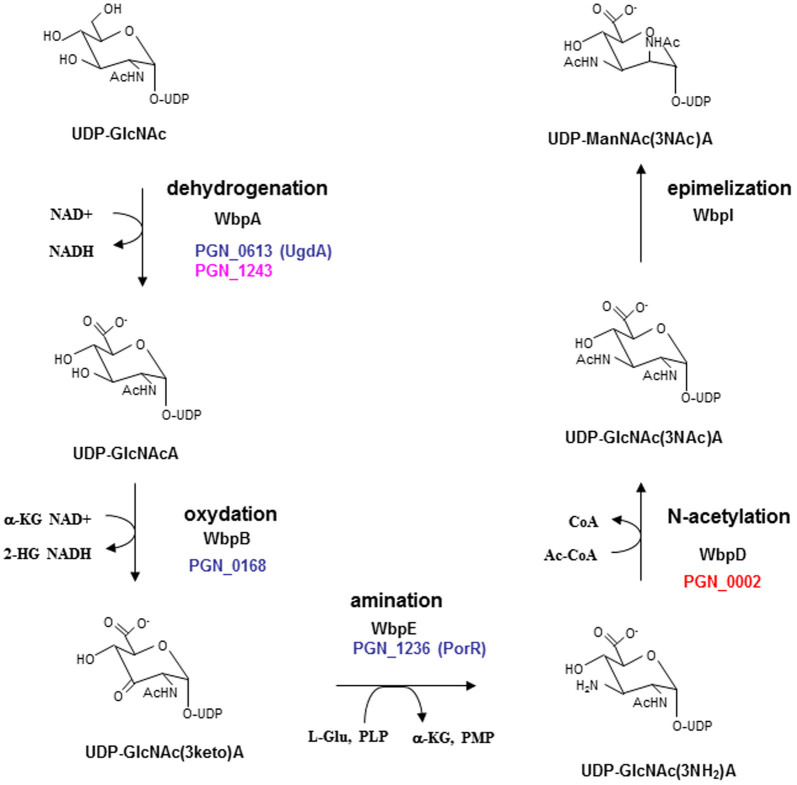
Wbp pathway for the biosynthesis of UDP-ManNAc(3NAc)A. Abbreviations were seen in Supplemental Text 3. Gene homologs of the Wbp pathway in *Porphyromonas gingivalis* are represented in colored PGN_ numbers.

We have recently shown that HG66 possesses O-LPS, but not A-LPS^13^. When expressed in a wild-type background, *porR* (PGN_1236), *vimA* (PGN_1056), *vimE* (PGN_1055), *wbpB* (PGN_0168), *ugdA* (PGN_0613), and *wbaP* (PGN_1896) mutants retain O-LPS but lose A-LPS^13^,^14^,^17^. We thus hypothesized that mutation of one or more of these genes was responsible for the A-LPS deficient phenotype in strain HG66. Among the above genes, we found that *wbpB* from strain ATCC 33277 conferred the pigmentation phenotype on strain HG66 (Figure 1A). HG66 cells expressing the *wbpB* gene from ATCC 33277 showed greater hemagglutination and gingipain activities on the cell surface compared with strain HG66 (Figure 1B, C). We have recently shown that cell lysates from strain HG66 exhibit no diffuse HBP35 and Rgp bands on a gel due to a lack of A-LPS^13^. Immunoblot analyses revealed that HG66 cell lysates expressing *wbpB* from ATCC 33277 contained A-LPS when probed with an anti-A-LPS antibody. The lysates also exhibited the diffuse HBP35 and Rgp bands that are also observed in the wild-type ATCC 33277 or W83 strains (Figure 2A). Interestingly, an anti-O-LPS antibody recognized higher molecular mass immunoreactive products in the HG66 cells expressing *wbpB* from ATCC 33277 compared with strain HG66 (Figure 2A). We next confirmed that purified LPS from HG66 cells expressing *wbpB* from ATCC 33277 contained both A-LPS and O-LPS by immunoblot analyses (Figure 2B). These results suggested that strain HG66 might encode a non-functional *wbpB* gene. Therefore, the coding sequence of the *wbpB* gene from strain HG66 was amplified by PCR and sequenced. We found that a nonsense mutation occurred at amino acid Q^240^ of the WbpB protein (Figure 2C, Supplemental Text 2). WbpB is an oxidoreductase that forms UDP-GlcNAc(3 keto)A from UDP-GlcNAcA (Figure 3). Additionally, WbpB is a component of the Wbp pathway, which synthesizes UDP-ManNAc(3NAc)A, a precursor of the ManNAc(3NAc)A residue in the B-band O-antigen of *P. aeruginosa*^33^,^34^. The *P. gingivalis* WbpB protein had high similarity to *P. aeruginosa* WbpB and *Bordetella pertussis* WlbA (Supplemental Figure 1). A truncated WbpB protein lacking C-terminal 83 amino acids is likely to be non-functional, although we did not confirm expression at the mRNA or protein levels.

### Genes in the Wbp pathway are involved in the biosynthesis of A-LPS

**Figure 4:**
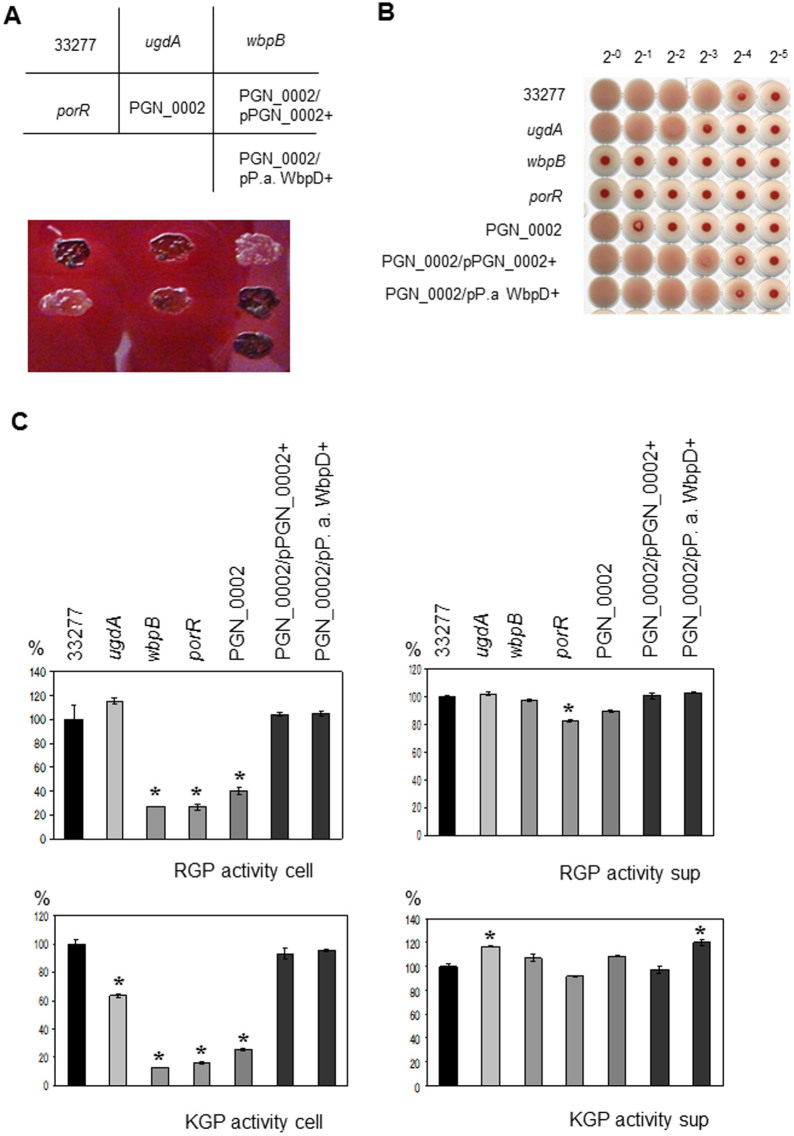
Pigmentation and hemagglutination and gingipain activities of various *Porphyromonas gingivalis* strains. (A) Colony pigmentation of *Porphyromonas gingivalis* cells on blood agar plates. (B) The hemagglutination activities of *P. gingivalis* strains were measured. (C) The Kgp and Rgp activities of the cell lysates (cell) and vesicle-containing culture supernatants (sup) of ATCC 33277 (wild-type), *ugdA*, *wbpB*, *porR*, PGN_0002, PGN_0002/PGN_0002+ and PGN_0002/*Pseudomonas aeruginosa* (P. a) WbpD+ were measured. The mean of each protease activity of ATCC 33277 was regarded as 100%. Asterisks indicate significant differences in enzymatic activity between ATCC 33277 and various strains (*P* < 0.01). The bars are expressed as the means ± standard deviation for triplicate samples from one of two independent experiments.

**Figure 5:**
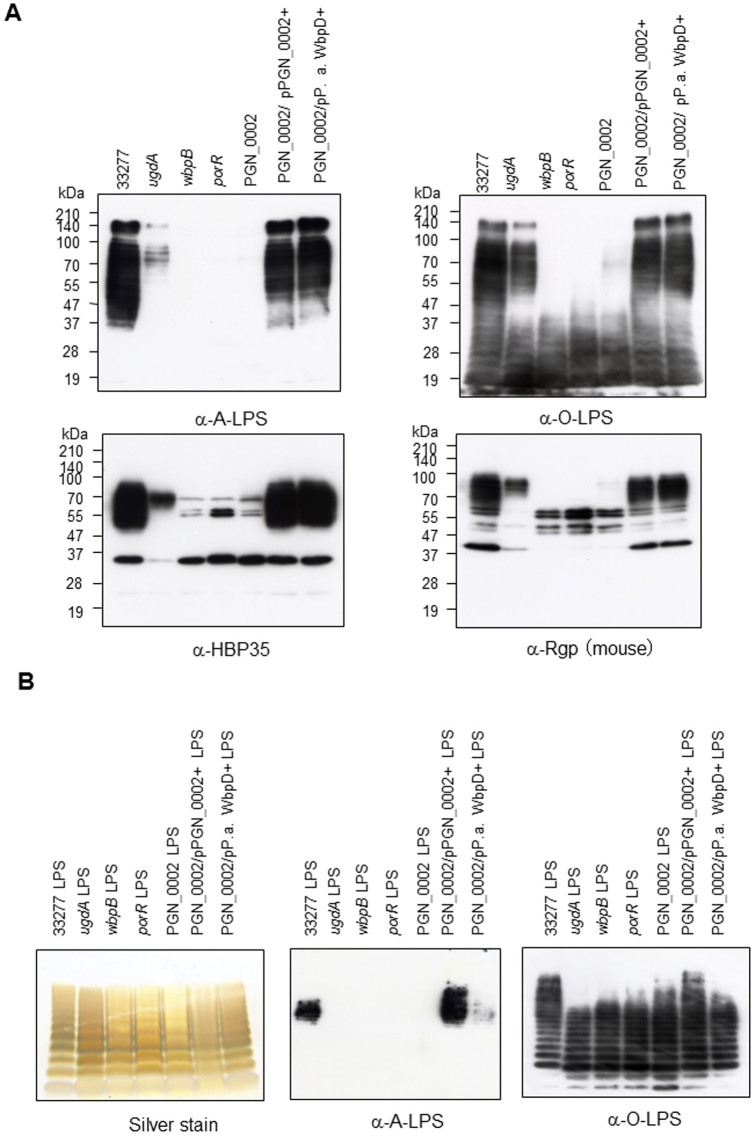
Immunoblot and LPS analyses of various *Porphyromonas gingivalis* strains. (A) Immunoblot analyses of cell lysates from various *P. gingivalis* strains were performed with various antibodies. (B) The LPS fraction from various *P. gingivalis* strains was stained by silver staining. Immunoblot analyses were performed with antibodies for LPS.

As WbpB belongs to the Wbp pathway, we next hypothesized that the nucleotide-activated sugar formed by the Wbp pathway in *P. gingivalis* was recognized by an anti-A-LPS antibody. Genomic analysis revealed that PGN_1243 and PGN_0631 (*ugdA*) were WbpA homologs, PGN_0168 (*wbpB*) was a WbpB homolog, PGN_1236 (*porR*) was a WbpE homolog, and PGN_0002 was a WbpD homolog; however, no WbpI homologs were found (Supplemental Figures 1, 2, 3, 4). It has been shown that the *ugdA*, *wbpB* and *porR* gene products are involved in the biosynthesis of A-LPS^4^,^16^,^17^. We focused on the PGN_0002 protein because it may be the last enzyme of the Wbp pathway in *P. gingivalis*, and it has not yet been characterized. PGN_0002 showed high similarity to *P. aeruginosa* WbpD and *Bordetella pertussis* WlbB, and both of these proteins possessed acetyltransferase activity, allowing the formation of UDP-GlcNAc(3NAc)A from UDP-GlcNAc(3NH_2_)A (Supplemental Figure 4). We constructed full PGN_1243 and PGN_0002 gene deletions; we were able to generate a PGN_0002 mutant, but not a PGN_1243 mutant. The PGN_0002 mutant exhibited a pigment-less phenotype and reduced hemagglutination and gingipain activities on the cell surface compared with wild-type cells (Figure 4A, B, C). Complementation of the PGN_0002 gene or expression of *P. aeruginosa* *wbpD* restored the pigmentation phenotype and, hemagglutination and gingipain activities on the cell surface (Figure 4A, B, C). Immunoblot analyses revealed that the *wbpB*, *porR* and PGN_0002 mutants were not immunoreactive to anti-A-LPS, and no HBP35 and Rgp diffuse bands were observed (Figure 5A). Interestingly, anti-O-LPS immunoreactive bands in the *wbpB*, *porR* and PGN_0002 mutants exhibited a lower molecular mass than those of the wild-type cells. The *ugdA* mutant showed decreased immunoreactivity to anti-A-LPS and reduced HBP35 and Rgp diffuse bands. The PGN_1243 gene products may compensate for the WbpA-like activity in the *ugdA* mutant. We also confirmed that purified LPSs from various *P. gingivalis* mutants in the Wbp pathway possessed O-LPS, but lacked A-LPS (Figure 5B).

## Discussion

Our previous study revealed that CTD proteins, which contain a conserved C-terminal domain, are transported to the cell surface via the type IX secretion system^6^. *P. gingivalis* has approximately 30 CTD proteins. Of these, 19 have been shown to be LPS-modified, suggesting that they bind to the A-LPS^35^. Specifically, RgpB, TapA and HBP35 have been experimentally shown to be anchored to the cell surface by binding to A-LPS^25^,^26^,^36^. As described above, *P. gingivalis* possesses two different LPS molecules, A-LPS and O-LPS. Recent studies have shown that 15 gene products play a role in the biosynthesis of A-LPS. Of these, the *wzy*, *waaL*, *gtfB* and *wzzP* gene products are involved in the biosynthesis of both A-LPS and O-LPS; mutations in these genes result in rough or semi-rough LPS^9^,^13^,^18^. Conversely, *porR* (PGN_1236), *vimA* (PGN_1056), *vimE* (PGN_1055), *wbpB* (PGN_0168), *ugdA* (PGN_0613) and *wbaP* (PGN_1896) mutants express O-LPS, but lack A-LPS, suggesting that some of these genes may be involved in A-LPS specific glycan synthesis. A-LPS consists of a lipid A-core and anionic polysaccharide (APS) repeating units containing phosphorylated branched mannan^10^,^11^. Paramonov et al.^10^ has demonstrated that the structure of APS is relatively similar to that of yeast mannan and is predicted to be synthesized by α1→6 and α1→2 mannosyltransferases. However, the genes encoding the mannosyltransferases have not been identified. Recently, the *vimF* gene product, which is one of proteins necessary for A-LPS biosynthesis, has been demonstrated to possess galactosyltransferase activity^22^. In addition, not all of the mannosidases are involved in A-LPS synthesis^37^. Therefore, the postulated structure of APS may not yet be fully understood.

**Figure 6:**
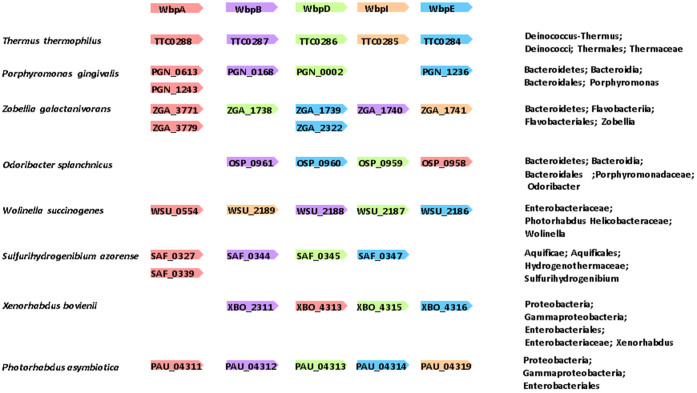
Gene context for representative gene homologs of the Wbp pathway in *Porphyromonas gingivalis* ATCC 33277, *Thermus thermophiles* HB27, *Zobellia galactanivorans*, *Odoribacter splanchnicus*, *Wolinella succinogenes*, *Sulfurihydrogenibium azorense*, *Xenorhabdus bovienii* and *Photorhabdus asymbiotica*. The lengths of the genes are not drawn to scale. Each homologous set of sequences is represented by one color.

In this study, we found that a *wbpB* gene mutation was responsible for the non-pigmented phenotype of strain HG66, and we further demonstrated that genes in the Wbp pathway were involved in the biosynthesis of A-LPS. The existence of ManNAc(3NAc)A was first reported in *P. aeruginosa* LPS more than 30 years ago^38^. It has been recently shown that the ManNAc(3NAc)A in *P. aeruginosa* is enzymatically synthesized by WbpA, WbpB, WbpE, WbpD and WbpI, proteins that together have been designated the Wbp pathway^33^,^34^,^39^. *Bordetella pertussis* has a similar Wbp pathway and possesses two *wbpA* gene homologs. These homologs are present separately and apart from the other gene homologs located in the *wlb* gene cluster^40^. Almost all of the genes in the Wbp pathway are located within a gene cluster in *P. aeruginosa* and *B. pertussis*. Gene homologs of *wbpA*, *wbpB*, *wbpE* and *wbpD* were located separately in the *P. gingivalis* genome, and no *wbpI* gene homologs were found in the genome (Figure 6). ManNAc(3NAc)A, the C2-epimer of GlcNAc(3NAc)A, is present not only in the LPS of *P. aeruginosa* and *B. pertussis*, but also in the cell wall polysaccharide of the Gram-positive thermophile, *Bacillus stearothermophilus*^41^. GlcNAc(3NAc)A has been reported to be present in the LPS of a number of *P. aeruginosa* strains, including P1-III and P14, the *N*-linked glycans of the methanogenic archaea, *Methanococcus voltae* and *Methanococcus maripalidus*, and the cell surface polysaccharide of the Gram-negative spirochete, *Treponema medium*^42^,^43^,^44^,^45^. A partial genome research survey revealed that *Thermus thermophiles* (*Deinococcus-Thermus*), *Zobellia galactanivorans* (*Bacteroidetes*), *Wolinella succinogenes* (*Epsilonproteobacteria*) and *Photorhabdus asymbiotica* (*Gammaproteobacteria*) possess 5 genes in the Wbp pathway. Conversely, *P. gingivalis* and *Odoribacter splanchnicus* (*Bacteroidetes*), *Sulfurihydrogenibium azorense* (*Aquificae*) and *Xenorhabdus bovienii* (*Gammaproteobacteria*) possess 4 genes in the Wbp pathway and lack the *wbpI* gene (Figure 6). The presence of 5-Wbp pathway gene homologs suggests that a particular species may utilize ManNAc(3NAc)A as a structural sugar, whereas the presence of 4-Wbp pathway gene homologs (lacking *wbpI*) suggests that a particular species may utilize GlcNAc(3NAc)A as a structural sugar. As ManNAc(3NAc)A and GlcNAc(3NAc)A have C2- and, C3-acetylated and C6-uronic acid forms, they are called di-acetylated mannuronic acid and glucuronic acid, respectively. Considering the conservation and the distribution of these molecules from archaea to Gram-positive and Gram-negative bacteria, di-acetylated uronic acid(s) may be structurally advantageous to endure a variety of environmental stresses.

It has been shown that O-glycosylated proteins are widely distributed in bacteria within the *Bacteroidetes* phylum^46^. Coyne et al.^46^ generated a specific antibody against the sugar portion of the O-glycosylated proteins in *Bacteroides fragilis*. Although this antibody mainly recognized protein bands from *Bacteorides* species, it also recognized a few *P. gingivalis* protein bands. To date, OMP85, Mfa1, PGN_0743 (a probable FKBP PPIase), PGN_0876 (a TPR domain protein), PGN_1513 (a hypothetical protein) and PGN_0729 (an outer membrane protein 41 precursor) have been reported to be glycoproteins in *P. gingivalis*^47^,^48^,^49^. As these glycoproteins each exhibit discrete bands in SDS-PAGE gels, they may be glycosylated by commonly known O-linked or N-linked glycosylation systems. Conversely, A-LPS-modified CTD proteins are expressed via the T9SS and are A-LPS dependent. However, the exact binding mechanism between CTD proteins and A-LPS is not known.

Based on the previously known molecules involved in the biosynthesis of A-LPS, we identified the gene mutation responsible for the non-pigmented phenotype of strain HG66 and found that genes in the Wbp pathway were involved in the biosynthesis of A-LPS. To date, the presence of di-acetylated glucuronic acid in *P. gingivalis* LPS molecules has not been documented. The analysis of di-acetylated glucuronic acid will shed light on novel features of the T9SS-dependent glycosylation system.

## Methods

### Bacterial strains and plasmids

The bacterial strains and plasmids used in this study are listed in Supplemental Tables 1 and 2, respectively^50^,^51^,^52^.

### Media and conditions for bacterial growth

*P. gingivalis* strains were grown anaerobically (80% N_2_, 10% CO_2_, 10% H_2_) in enriched brain-heart infusion (BHI) broth (Becton Dickinson, Franklin Lakes, NJ) or on enriched tryptic soy (TS) agar plates (Nissui, Tokyo, Japan) supplemented with 5 μg/ml hemin (Sigma, St. Louis, MO) and 0.5 μg/ml menadione (Sigma). For blood agar plates, defibrinated laked sheep blood was added to enriched tryptic soy agar at 5%. Luria-Bertani (LB) broth and LB agar plates were used for growth of *E. coli* strains. Antibiotics were used at the following concentrations: ampicillin (Ap; 10 μg/ml for *P. gingivalis*, 100 μg/ml for *E. coli*), erythromycin (Em; 10 μg/ml for *P. gingivalis*), gentamycin (Gm; 50 μg/ml for *P. gingivalis*) and tetracycline (Tc; 0.7 μg/ml for *P. gingivalis*).

### Chemicals

The proteinase inhibitors Nα-p-tosyl-_L_-lysine chloromethyl ketone (TLCK) and iodoacetamide were purchased from Wako (Japan), and leupeptin was obtained from the Peptide Institute (Japan).

### Construction of *P. gingivalis* strains

The oligonucleotides used in this study are listed in Supplemental Table 3. The general manipulation of DNA, restriction and mapping of plasmids and transformation of *E. coli* and *P. gingivalis* have been described in detail elsewhere^26^. The chromosomal DNA from *P. gingivalis* ATCC 33277 was used as the template for cloning purposes. The construction of various mutants from *P. gingivalis* ATCC 33277 or complemented strains from the mutants is described in Supplemental Text 1.

### Sequencing of the *wbpB* gene of *P. gingivalis* HG66

The coding region of the *wbpB* gene from strain HG66 was amplified using PGN_0168upFw/PGN_0168upRev primers and cloned into the pGEM-T Easy vector. Sequencing was performed by direct sequencing using the purified PCR product or by the common method using the cloned vector with SP6/T7 primers.

### Enzymatic assay

Kgp and Rgp activities were determined using the synthetic substrates benzyloxycarbonyl-l-histidyl-l-glutamyl-l-lysine-4-methyl-coumaryl-7-amide (Z-His-Glu-Lys-MCA) and benzyloxycarbonyl-l-phenyl-l-arginine-4-methyl -coumaryl-7-amide (Z-Phe-Arg-MCA), respectively (both from Peptide Institute, Japan). In brief, appropriate amounts of the bacterial cell as well as the bacterial culture supernatant, were added to the reaction mixture (0.25 ml) containing 5 mM cysteine, 20 mM sodium phosphate buffer, pH 7.5, and 10 μM each fluorogenic substrate. After 10 min incubation at 40°C, the reaction was terminated by adding 100 mM sodium acetate buffer, pH 5.0, containing 10 mM iodoacetic acid (0.25 ml). The released 7-amino-4-methyl-coumarine was measured at 460 nm (excitation at 380 nm) by fluorescence spectrophotometer Beckman Coulter DTX 800 (Brea, CA).

### Hemagglutination activity

Overnight cultures of *P. gingivalis* strains in enriched BHI medium were centrifuged, washed once with phosphate-buffered saline (PBS), and suspended in PBS at an optical density of 1.0 at 595 nm. The bacterial suspensions were then diluted in a two-fold series with PBS. A 100-μl aliquot of each suspension was mixed with an equal volume of defibrinated sheep erythrocyte suspension (1% in PBS) and incubated in a round-bottom microtiter plate at room temperature for 3 h.

### Preparation of *P. gingivalis* LPS

A fully grown 200-ml culture was centrifuged, washed once with distilled water and suspended with 5 ml of 10 mM Tris-HCl (pH8.0) containing 2% SDS. Next, 20 μg/ml of DNase and RNase (Sigma, St. Louis, MO. USA) was added at 37°C for 1 h, and 20 μg/ml of proteinase K (Takara, Japan) was added at 37°C overnight. After the overnight incubation, the samples were mixed with preheated 90% phenol at 68°C and stirred at 68°C for 20 min. The samples were centrifuged at 7,000 rpm for 20 min. The aqueous solutions were dialyzed against milliQ water to remove residual phenol. The dialyzed solutions were centrifuged at 100,000 × *g* for 3 h, and the precipitated samples were dissolved in milliQ water as the LPS fraction. LPS was visualized by silver staining.

### Gel electrophoresis and immunoblot analysis

SDS-PAGE and immunoblot analysis were performed as described previously^13^,^19^,^26^. For visualization of LPS, modified SDS-PAGE containing 4 M urea in the separating gel or Tris-Tricine SDS-PAGE was used.

### Preparation of antiserum

An anti-HBP35 rabbit polyclonal antibody^53^ was used to detect HBP35, mAb 1B5 was used to detect A-LPS^12^, mAb TDC-5-2-1 was used to detect O-LPS and mouse polyclonal antiserum against the amino acid region (E^361^-L^375^) within the catalytic domain of RgpB (PGN_1416) was used to detect Rgp^13^. To prepare rabbit antiserum against the amino acid region (E^361^-L^375^) within the catalytic domain of RgpB (PGN_1416), a rabbit was immunized by EveBioscience Co., Ltd. (Wakayama, Japan), and the antiserum was named anti-Rgp (rabbit) to distinguish it from anti-Rgp (mouse) described above.

### Statistical analysis

The significance of all described comparisons was established using two-tailed unpaired *t* tests on triplicate samples with a significance level of 0.01.

## Author Contributions

M.S. and K.N. conceived and designed the experiments. M.S. performed the experiments. M.S. and K.N. analysed the data. K.S., H.Y. and M.N. contributed reagents/materials/analysis tools. M.S. and K.N. wrote the manuscript.

## Supplementary Material

Supplementary Information Supplemental Information
