# Supplementary material for: Involvement of the Wbp pathway in the biosynthesis of Porphyromonas gingivalis lipopolysaccharide with anionic polysaccharide
Source: Sci Rep. 2014 May 23;4:5056. doi: 10.1038/srep05056 (PMC4031482; doi:10.1038/srep05056)
Supplement: Supplementary Information — Supplemental Information [file srep05056-s1.pdf]

Supplementary Information for:

Involvement of the Wbp pathway in the biosynthesis of *Porphyromonas gingivalis* lipopolysaccharide with anionic polysaccharide

Mikio Shoji[1], Keiko Sato[1], Hideharu Yukitake[1], Mariko Naito[1] and Koji Nakayama[1]

[1] Division of Microbiology and Oral Infection, Department of Molecular Microbiology and Immunology, Nagasaki University Graduate School of Biomedical Sciences, Nagasaki, 852-8588, Japan

Correspondence and requests for materials should be addressed to M. S.

(m-shoji@nagasaki-u.ac.jp)

Supplemental Figure 1 | Comparison of amino acid sequences among WbpB homologs. *Porphyromonas gingivalis* ATCC 33277: PGN_0168, *Pseudomonas aeruginosa* PAO1: PA3158, *Helicobacter pylori* NCTC 11637: HP0679 and *Bordetella pertussis* Tohama I: BP0093 were compared. The alignment was created using the ClustalW and Boxshade programs hosted at the Swiss EMBnet web site. The red triangle indicates the nonsense mutation in *P. gingivalis* strain HG66.

Supplemental Figure 2 | Comparison of amino acid sequences among WbpA homologs. *Porphyromonas gingivalis* ATCC 33277: PGN_0613, PGN_1243, *Pseudomonas aeruginosa* PAO1: PA3159 and *Bordetella pertussis* Tohama I: BP1629, BP3150 were compared. The alignment was created using the ClustalW and Boxshade programs hosted at the Swiss EMBnet web site.

Supplemental Figure 3 | Comparison of amino acid sequences among WbpE homologs. *Porphyromonas gingivalis* ATCC 33277: PGN_1236, *Pseudomonas aeruginosa* PAO1: PA3155 and *Bordetella pertussis* Tohama I: BP0091 were compared. The alignment was created using the ClustalW and Boxshade programs hosted at the Swiss EMBnet web site. The red triangle indicates the essential residue for WbpE activity.

Supplemental Figure 4 | Comparison of amino acid sequences among WbpD homologs. *Porphyromonas gingivalis* ATCC 33277: PGN_0002, *Pseudomonas aeruginosa* PAO1: PA3156 and *Bordetella pertussis* Tohama I: BP0092 were compared. The alignment was created using the ClustalW and Boxshade programs hosted at the Swiss EMBnet web site. The red triangles indicate the essential residue for WbpD activity.

Supplemental Table 1 | Strains used in this study.

Supplemental Table 2| Plasmids used in this study.

Supplemental Table 3| Primers used in this study.

Supplemental Text 1| Construction of *P. gingivalis* mutant strains.

Construction of *P. gingivalis* mutants

To create *P. gingivalis* PGN_0168 (*wbpB*)::Em^r (KDP220) and PGN_0002::Em^r (KDP222) mutants, the upstream regions were amplified with PGN_0168upFw/PGN_0168upRev and PGN_0002upFw/PGN_0002upRev primers, respectively, using *P. gingivalis* ATCC 33277 as the template, and cloned into the pCR4 vector or pGEM-T Easy vector. Each upstream and downstream region was swapped with the appropriate restriction region of pKD740 [26], generating pKD892 and pKD893. The resulting targeting vectors pKD892 and pKD893 were linearized with SacI and introduced into *P. gingivalis* ATCC 33277 by electroporation followed by selection on blood agar plates containing Em (10 μg/ ml).

To create a *P. gingivalis* PGN_1236 (*porR*)::Tc^r (KDP221) mutant, the upstream region was amplified with PGN_1236upFw/PGN_1236upRev primers using *P. gingivalis* ATCC 33277 as the template and cloned into the pCR4 vector, yielding pCR4-porRup. The downstream region was amplified with PGN_1236dwFw/PGN_1236dwRev primers using *P. gingivalis* ATCC 33277 as the template and cloned into the pCR4 vector, yielding pCR4-porRdw1. To eliminate the EcoRI site that was closest to the NotI site in pCR4-porRdw1, pCR4-porRdw1 was digested with NotI and was then self-ligated, yielding pCR4-porRdw2. The EcoRI-BamHI region of pCR4-porRup was inserted into the same region of pCR4-porRdw2, yielding pCR4-porRupdw2. The *tetQ* gene, which is the BamHI-BglII region of pKD375 [51], was inserted into the BamHI site of pCR4-porRupdw2, generating pKD894. The resulting targeting vector pKD894, linearized with NotI, was introduced into *P. gingivalis* ATCC 33277 by electroporation and was then selected on blood agar plates containing Tc (0.7 μg/ ml).

Construction of *P. gingivalis* complemented strains

The promoter region of the *Porphyromonas gulae* catalase gene (accession no. AB083039 in GenBank/EMBL/DDBJ databases) was amplified using p6-34-F-KpnI/p6-34-R-SalI from pKD955 [6] and was cloned into the pCR4 vector to yield pCR4-cat pro. A KpnI-EcoRI DNA fragment containing the promoter region of the *Porphyromonas gulae* catalase gene of pCR4-cat pro was swapped into the same region of pKD854 [5] to yield pBSSK-cat pro-rgpBter.

To create complementation plasmids for the *wbpB* (PGN_0168) and PGN_0002 genes, *P. gingivalis* ATCC 33277 was used as the template for amplification with PGN_0168compFw/PGN_0168compRev or PGN_0002compFw/PGN_0002compRev primers, respectively. Each PCR product was cloned into pUC118, yielding pUC118-*wbpB* and pUC118-PGN_0002.

To create a complementation plasmid with the *wbpD* gene from *Pseudomonas aeruginosa*, the coding region of *wbpD* was amplified from *P. aeruginosa* using the *P. a. wbpD*Fw/ *P. a. wbpD*Rev primers. The PCR product was cloned into pUC118, yielding pUC118-*P.a. wbpD*.

The SalI-XbaI DNA fragment containing the coding region of the *wbpB*, PGN_0002 and *P.a. wbpD* genes from pUC118-*wbpB*, pUC118-PGN_0002 and pUC118-*P. a. wbpD* was inserted into the same region of pBSSK-cat pro-rgpBter to yield pBSSK-cat pro-*wbpB*-rgpBter, pBSSK-cat pro-PGN_0002-rgpBter and pBSSK-cat pro-*P.a. wbpD*-rgpBter. The KpnI-NotI DNA fragment of pBSSK-cat pro-*wbpB*-rgpBter, pBSSK-cat pro-PGN_0002-rgpBter and pBSSK-cat pro-*P.a. wbpD*-rgpBter was inserted into the same region of a pTCB vector [52] to yield pTCB-*wbpB*, pTCB-PGN_0002 and pTCB-*P.a. wbpD*. The pTCB vector containing *wbpB*, PGN_0002 or *P. a wbpD* was introduced into *Escherichia coli* S17-1 [53] by electroporation and was then selected on LB agar plates containing Ap (100 μg/ ml). The

transformant was mated with strain HG66 or the PGN_0002 mutant (KDP222) and was then selected on blood agar plates containing Gm (50 μg/ ml) and Tc (0.7 μg /ml), yielding HG66/pWbpB+, PGN_0002/pPGN_0002+ (KDP223) and PGN_0002/p*P. a*.WbpD+ (KDP224).

Supplemental Text 2| Sequence comparison of the *wbpB* gene in *P. gingivalis* strains.

```
                   10         20         30         40         50
W83        ATGCACAACT TTGCCATCAT CGGTGTGGCC GGCTATATTG CTCCGCGCCA
TDC60      ATGCACAACT TTGCCATCAT CGGTGTGGCC GGCTATATTG CTCCGCGCCA
HG66       ATGCACAACT TTGCCATCAT CGGTGTGGCC GGCTATATTG CTCCGCGCCA
33277      ATGCACAACT TTGCCATCAT CGGTGTGGCC GGCTATATTG CTCCGCGCCA
Consensus  ********** ********** ********** ********** **********
                   60         70         80         90        100
W83        CCTTCGCGCC ATCAAGGATA CAGGCAACCG CTTGGTATCG GCTATGGATA
TDC60      CCTTCGCGCC ATCAAAGATA CAGGCAATCG CTTGGTATCG GCTATGGATA
HG66       CCTTCGCGCC ATCAAGGATA CAGGCAACCG CTTGGTATCG GCTATGGATA
33277      CCTTCGCGCC ATCAAGGATA CAGGCAACCG CTTGGTATCG GCTATGGATA
Consensus  ********** ***** **** ******* ** ********** **********
                  110        120        130        140        150
W83        AGTTCGACAG TGTGGGTATC ATGGACAGTT ACTTCCCGAA AGCTGCCTTC
TDC60      AGTTCGACAG TGTGGGTATC ATGGACAGTT ACTTCCCGAA AGCTGCCTTC
HG66       AGTTCGACAG TGTGGGTATC ATGGACAGTT ACTTCCCGAA AGCTGCCTTC
33277      AGTTCGACAG TGTGGGTATC ATGGACAGTT ACTTCCCGAA AGCTGCCTTC
Consensus  ********** ********** ********** ********** **********
                  160        170        180        190        200
W83        TTTACGGAGC AAGAACTGTT CGACCGTCAC AACTCCAAGC TACGAGGAAC
TDC60      TTTACGGAGC AAGAACTGTT CGACCGTCAC AACTCCAAGC TACGAGGAAC
HG66       TTTACGGAGC AAGAACTGTT CGATCGTCAC AACTCCAAGC TACGAGGAAC
33277      TTTACGGAGC AAGAACTGTT CGATCGTCAC AACTCCAAGC TACGAGGAAC
Consensus  ********** ********** *** ****** ********** **********
                  210        220        230        240        250
W83        CGATCAGGCT ATCGACCACG TGTCCGTTTG TACACCGAAC TATCTGCACG
TDC60      CGATCAGGCT ATCGACCATG TGTCCGTTTG TACACCGAAC TATCTGCACG
HG66       CGATCAGGCT ATCGACCACG TGTCCGTTTG TACACCGAAC TATCTGCACG
33277      CGATCAGGCT ATCGACCACG TGTCCGTTTG TACACCGAAC TATCTGCACG
Consensus  ********** ******** * ********** ********** **********
```

```
                    260       270       280       290       300
W83          ATGCTCATTG TCGCTATGGC CTGCGACTTG GAGCGGATGT GATATGCGAG
TDC60        ATGCTCATTG TCGCTATGGC CTACGACTTG GAGCGGATGT GATATGCGAG
HG66         ATGCTCATTG TCGCTATGGC CTGCGACTTG GAGCGGATGT GATATGCGAG
33277        ATGCTCATTG TCGCTATGGC CTGCGACTTG GAGCGGATGT GATATGCGAG
Consensus    ********** ********** ** ******* ********** **********
                    310       320       330       340       350
W83          AAGCCTTTGG TGCTCAATCC TTGGAATATA GACGCTTTGC AGGAGATCGA
TDC60        AAGCCTTTGG TGCTCAATCC TTGGAATATA GACGCTTTGC AGGAGATCGA
HG66         AAGCCTTTGG TGCTCAATCC TTGGAATATA GACGCTTTGC AGGAGATCGA
33277        AAGCCTTTGG TGCTCAATCC TTGGAATATA GACGCTTTGC AGGAGATCGA
Consensus    ********** ********** ********** ********** **********
                    360       370       380       390       400
W83          GCGAGAGACG GGGCATCGGA TCTATAATAT TCTTCAACTG CGACTCCATG
TDC60        GCAAGAGACG GGGCATCGGA TCTATAATAT TCTTCAACTG CGACTCCATG
HG66         GCAAGAGACG GGGCATCGGA TCTATAATAT TCTTCAACTG CGACTCCATG
33277        GCAAGAGACG GGGCATCGGA TCTATAATAT TCTTCAACTG CGACTCCATG
Consensus    ** ******* ********** ********** ********** **********
                    410       420       430       440       450
W83          AGTCGATCGT GGCCCTGAAG AGAAAGATCG AATCCGGATC GGCCGACAAG
TDC60        AGTCTATCGT GGCCCTGAAG AGAAAGATCG AATCCGGACC GGCCGACAAG
HG66         AGTCTATCGT GGCCCTGAAG AGAAAGATCG AATCCGGACC GGCCGACAAG
33277        AGTCTATCGT GGCCCTGAAG AGAAAGATCG AATCCGGACC GGCCGACAAG
Consensus    **** ***** ********** ********** ******** * **********
                    460       470       480       490       500
W83          ATCTATGATG TCACGCTTAC ATATATTACT TCGCGCGGCA ATTGGTACTA
TDC60        ATCTATGATG TCACGCTGAC GTATATTACT TCGCGCGGCA ATTGGTACTA
HG66         ATCTATGATG TCACGCTGAC GTATATTACT TCGCGTGGCA ACTGGTACTA
33277        ATCTATGATG TCACGCTGAC GTATATTACT TCGCGTGGCA ACTGGTACTA
Consensus    ********** ******* **  ********* ***** **** * ********
                    510       520       530       540       550
W83          TACGAGCTGG AAGGGAGACG AGCGTAAGAG CGGTGGCATA GCTACGAACA
TDC60        TACGAGCTGG AAGGGAGACG AGCGTAAGAG CGGTGGCATA GCTACGAACA
HG66         TACGAGCTGG AAGGGAGACG AGCGTAAGAG CGGCGGTATA GCAACGAATA
33277        TACGAGCTGG AAGGGAGACG AGCGTAAGAG CGGCGGTATA GCAACGAATA
Consensus    ********** ********** ********** *** ** *** ** ***** *
```

```
                    560        570        580        590        600
W83          TCGGTGTTCA TTTCTACGAC ATGCTGTCAT GGATTTTCGG CAGTGTACGA
TDC60        TCGGTGTTCA TTTCTACGAC ATGCTGTCAT GGATTTTCGG CAGTGTACGA
HG66         TCGGTGTCCA TTTCTACGAC ATGCTGTCGT GGATTTTCGG CAGTGTCCGA
33277        TCGGTGTCCA TTTCTACGAC ATGCTGTCGT GGATTTTCGG CAGTGTCCGA
Consensus    ******* ** ********** ******** * ********** ****** ***
                    610        620        630        640        650
W83          CACAATGTCG TACATGTGTG TGTGCATGAC AGGGCTGCCG GCTATTTGGA
TDC60        CACAATGTCG TACATGTGTG TGTGCATGAC AGGGCTGCCG GCTATTTGGA
HG66         CACAATGTCG TACATGTGTA TGAGCATGAC AGGGCTGCCG GCTATCTGGA
33277        CACAATGTCG TACATGTGTA TGAGCATGAC AGGGCTGCCG GCTATCTGGA
Consensus    ********** *********  ** ******* ********** ***** ****
                    660        670        680        690        700
W83          ATTGGAGCGC GCCCGTGTGC GTTACTTCCT TTCGATCAAT GAGGAGCTTC
TDC60        ATTGGAGCGC GCCCGTGTGC GTTACTTCCT TTCGATCAAT GAGGAGCTTC
HG66         ATTGGAGCGC GCCCGTGTGC GTTACTTCCT CTCGATCAAT GAAGAGCTTC
33277        ATTGGAGCGC GCCCGTGTGC GTTACTTCCT CTCGATCAAT GAAGAGCTTC
Consensus    ********** ********** **********  ********* ** *******
                    710        720        730        740        750
W83          TGCCCGAGAA TGCCGTACAG GGAGAGAAGC GAACCTTTCG TACGATAGAA
TDC60        TGCCCGAGAA TGCCGTACAG GGAGAGAAGC GAACCTTTCG TACGATAGAA
HG66         TGCCCGAGAA TGCCGTATAG GGAGAGAAGC GAACCTTTCG TACGATAGAA
33277        TGCCCGAGAA TGCCGTACAG GGAGAGAAGC GAACCTTTCG TACGATAGAA
Consensus    ********** ******* ** ********** ********** **********
                    760        770        780        790        800
W83          ATCGACGGCG AATCATTCGA GTTCAGTGAA GGGTTTACGG AGCTGCACAC
TDC60        ATCGACGGCG AATCATTCGA GTTCAGTGAA GGGTTTACGG AGCTGCACAC
HG66         ATCGACGGCG AATCATTCGA GTTCAGTGAA GGGTTTACGG AGCTGCACAC
33277        ATCGACGGCG AATCATTCGA GTTCAGTGAA GGGTTTACGG AGCTGCACAC
Consensus    ********** ********** ********** ********** **********_
                    810        820        830        840        850
W83          CGAGAGTTAT CGGCGGATTT TGGCCGGTGA AGGTTTCGGT TTGGACGAAG
TDC60        CGAGAGTTAT CGGCGGATTT TGGCCGGTGA AGGTTTCGGT TTGGACGAAG
HG66         CGAGAGTTAT CGGCGGATTT TGGCCGGTGA AGGTTTCGGT TTGGACGAAG
33277        CGAGAGTTAT CGGCGGATTT TGGCCGGTGA AGGTTTCGGT TTGGACGAAG
Consensus    ********** ********** ********** ********** **********
```

```
                    860        870        880        890        900
W83         CTCGCAACAG CATTCAGATC GTTCATGACA TTCGCAATGC TTCTCCCGTA
TDC60       CTCGCAACAG CATTCAGATC GTTCATGACA TTCGCAATGC TTCTCCCGTA
HG66        CTCGCAACAG CATTCAGATC GTTCATGACA TTCGCAATGC TTCTCCCGTA
33277       CTCGCAACAG CATTCAGATC GTTCATGACA TTCGCAATGC TTCTCCCGTA
Consensus   ********** ********** ********** ********** **********
                    910        920        930        940        950
W83         GGACTGAAGG GCGAATACCA CCCCCTTGCA CGTTTGCCCC TGGCCTCTCA
TDC60       GGACTGAAGG GCGAATACCA CCCCTTTGCA CGTTTGCCCC TGGCCTCTCA
HG66        GGACTGAAGG GCGAATACCA CCCCTTTGCA CGTTTGCCCC TGGCCTCTCA
33277       GGACTGAAGG GCGAATACCA CCCCTTTGCA CGTTTGCCCC TGGCCTCTCA
Consensus   ********** ********** **** ***** ********** **********
                    960        970
W83         TCCTTTCGGA TGGAGACGCT GA
TDC60       TCCTTTCGGA TGGAGACGCT GA
HG66        TCCTTTCGGA TGGAGACGCT GA
33277       TCCTTTCGGA TGGAGACGCT GA
Consensus   ********** ********** **
```

Yellow color indicates the same residues between HG66 and other strains.

Red color indicates the HG66 nonsense mutation.

Supplement Text 3| Abbreviations

GlcNAc, glucosamine; GlcNAcA, 2-acetamido-2,3-dideoxy-D-glucuronic acid; 3-keto-D-GlcNAcA, 2-acetamido-2-deoxy-D-ribo-hex-3-uluronic acid; GlcNAc3NA, 2-acetamido-3-amino-2,3-dideoxy-D-glucuronic acid; GlcNAc3NAcA, 2,3-diacetamido-2,3-dideoxy-D-glucuronic acid; ManNAc3NAcA, 2,3-diacetamido-2,3-dideoxy-D-mannuronic acid. AcCoA, acetyl-coenzyme A; CoA, coenzyme A; 2-HG, 2-hydroxyglutarate; α-KG, α-ketoglutarate; NAD^+, nicotinamide adenine dinucleotide; NADH, nicotinamide adenine dinucleotide hydrate; PLP, pyridoxal 5′-phosphate; PMP, pyridoxamine 5′phosphate.

Supplemental Figure1

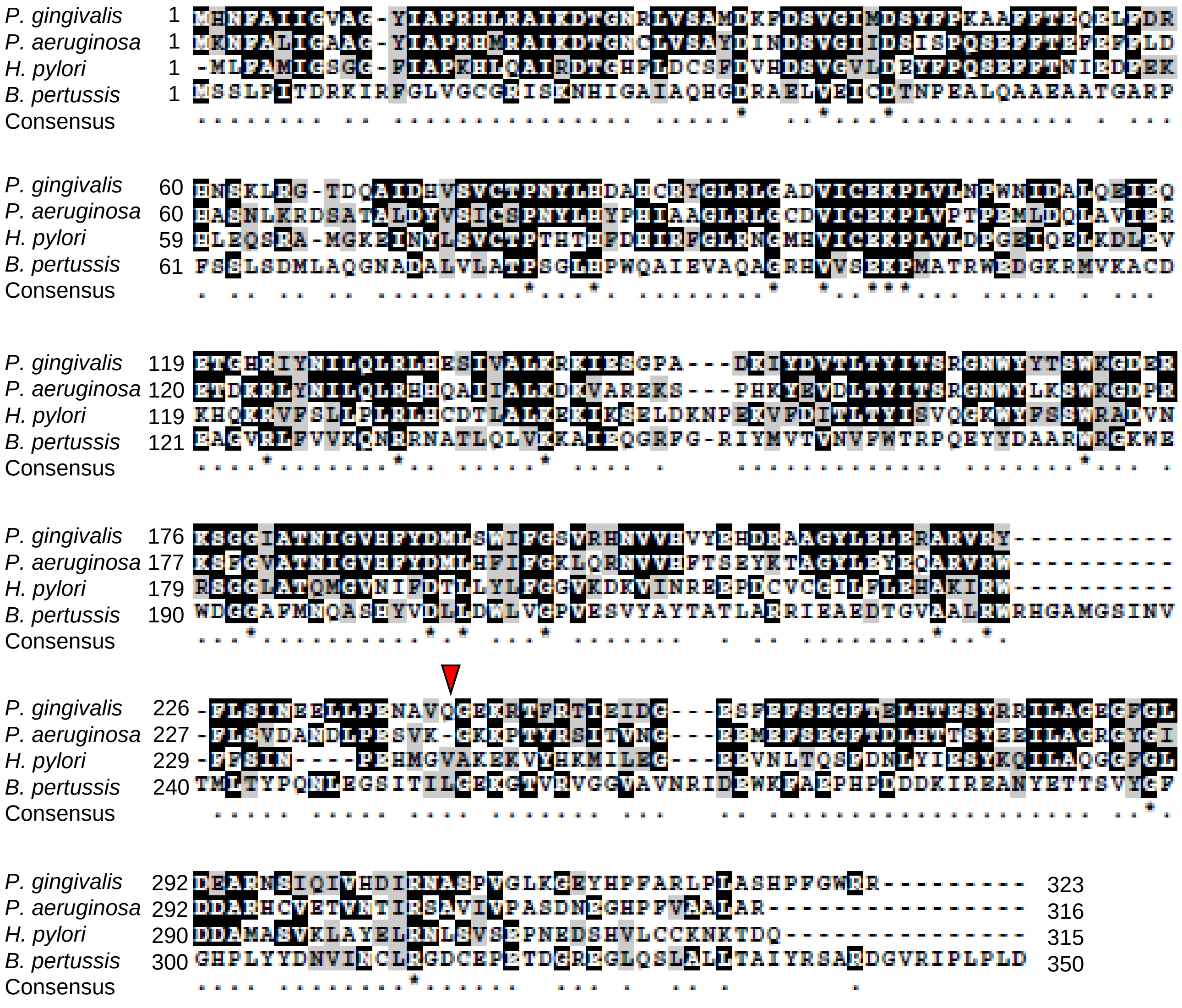

Supplemental Figure 2

```
BP1629       1  ------------------------------------------------------------
BP3150       1  ------------------------------------------------------------
PGN_0613     1  ------------------------------------------------------------
PA WbpA      1  ------------------------------------------------------------
PGN_1243     1  MGQACIGSRPISFLWEVRTCRRFIGSYTLIRRVIVSHVLSGSRHYIPATGRGNSRLSLDK

BP1629       1  --------MPKKFSNPADVHLAVVGLGYVGLPLAVEFGKK-RPVIGFDINERRIAALKAG
BP3150       1  -------------MRIDDVKLAVVGLGYVGLPLAVEFGKK-RPVIGFDINERRIAALKAG
PGN_0613     1  -MKPYKALTIINTKEMNNIRIAIIGLGYVGLPLARLFSTK-FPTIGFDINPNRVEELISG
PA WbpA      1  ---MIDVNTVVEKFKSRQALIGIVGLGYVGLPLMLRYNAIGFDVLGIDIDDVKVDKLNAG
PGN_1243    61  ASPFYLTFVPKNSISYYNMDIAVVGIGYVGLVSATCFAELGANVRCIDTDRNKIEQLNSG
Consensus                        .......*.*****.... .. .  .....*... ... * .*

BP1629      52  HDHTLEVEDDELARAAQLS---YTAER--EELGKANVFIVTVPTPIDEYKQPDLTPLVKA
BP3150      47  HDHTLEVEDDELARAAQLS---YTAER--EELGKANVFIVTVPTPIDEYKQPDLTPLVKA
PGN_0613    59  YDSTGEIGNELLQEALQKG---LLCTTKIEEIRDCNFYIVTVPTPIDHNHTPDLRPLLAA
PA WbpA     58  QCYIEHIPQAKIAKARASG---FEATTDFSRVSECDALILCVPTPLNKYREPDMSFVINT
PGN_1243   121  TIPIYEPGLEKMIARNVKAGRLRFGTEIEQAVPEADIIFIAVGTPAGEDGSADMSYVLDA
Consensus        . . ..  . .... . .   . ..   ... ........*.**..... .*..... .

BP1629     107  SETIGAVLKRGDIVIYESTVYPGATEEDCVPVLERVSGLKFNQDFYAGYSPERINPGDKA
BP3150     102  SETIGAVLKRGDIVIYESTVYPGATEEDCVPVLERVSGLKFNQDFYAGYSPERINPGDKA
PGN_0613   116  SETIGKVIDQGDVVVYESTVYPGVTEDECIPIIEKVSGLKYNVDFFAGYSPERINPGDKV
PA WbpA    115  TDALKPYLRVGQVVSLESTTYPGTTEEELLPRVQEG-GLVVGRDIYLVYSPEREDPGNPN
PGN_1243   181  ARSIGRAMSRYILIVTKSTVPVGSYRLIRKAIQEELDKREVLIDFDIASNPEFLKEGNAI
Consensus       ..... ...........**...* .................. *......**....*..

BP1629     167  HRVNTIKKVTSGS-TPEVAELVDQLYN-EIITAGTHKASSIRVAEAAKVIENTQRDVNIA
BP3150     162  HRVNTIKKVTSGS-TPEVAELVDQLYN-EIITAGTHKASSIRVAEAAKVIENTQRDVNIA
PGN_0613   176  YTVENIKKVTSGS-TPEIADYVDRMYN-TVLVNGTHKASSIKVAEASKIIENAQRDVNIA
PA WbpA    174  FETRTIPKVIGGH-TPQCLEVGIALYE-QAIDR-VVPVSSTKAAEMTKLLENIHRAVNIG
PGN_1243   241  DDFMKPDRVVVGVDSDRARELITSLYKPMLLNNFRVLFMDIASAEMTKYAANAMLATRIS
Consensus         . ....*..*. ......... .*.  ..  ..........**..*...* .....*.

BP1629     225  LINELALIFNKMGIDTEAVLQAAGTKWN--FLPFRPG-LVGGHCIGVDPYYLTHKAQAIG
BP3150     220  LINELALIFNKMGIDTEAVLQAAGTKWN--FLPFRPG-LVGGHCIGVDPYYLTHKAQAIG
PGN_0613   224  FMNEIAKIFNAMEIDTREVLEAAATKWN--FLPFQPG-LVGGHCIGVDPYYLIQRAQVYG
PA WbpA    231  LVNEMKIVADRMGIDIFEVVDAAATKPFG-FTPYYPGPGLGGHCIPIDPFYLTWKAREYG
PGN_1243   301  FMNDVANLCERVGADVSMVRLGIGSDSRIGSKFLYPGCGYGGSCFPKDVKALIRTAEDNG
Consensus       ..*...........*.  *. .......  .... ** ..**.*...*...*...*.  *

BP1629     282  YHPEIILAGRRLNDSMGGYVVSQLVKAMAKRRIHVEGARVLLMGLTFKENCPDLRNTRVV
BP3150     277  YHPEIILAGRRLNDSMGGYVVSQLVKAMAKRRIHVEGARVLLMGLTFKENCPDLRNTRVV
PGN_0613   291  VYPRVLMSARRLNDSMGSYIASQTIKQMNKAGIMVKDARILILGFTFKENCPDIRNTKVI
PA WbpA    290  LHTRFIELSGEVNQAMPEYVLGKLMDGLNEAGRALKGSRVLVLGIAYKKNVDDMRESPSV
PGN_1243   361  YRMEVLEAVERVNEKQKSILFDKFSTYYKGN---VQGRCVAIWGLSFKPGTDDMREAPSL
Consensus       ............*.... .......... .  . . .......*...*....*.*.....

BP1629     342  DIVRELGEYHVDVDVYDPWVDPAEAEHEYGITPVAKPAAGA----YDAIILAVSHHQFVE
BP3150     337  DIVRELGEYHVDVDVYDPWVDPAEAEHEYGITPVAKPAAGA----YDAIILAVSHHQFVE
PGN_0613   351  DTYSTLQEYTKNIIVHDPWANPSIAEQVYGIKIHNEFPKGK----FDAIIIAVAHRDFSN
PA WbpA    350  EIMELIEAKGGMVAYSDPHVPVFPKMREHHFELSSEPLTAENLARFDAVVLATDHDKFDY
PGN_1243   418  VLIEKLLEVGCRVRVYDPVAMKEAQKRLGDKVEYTTDMYDAVRG-AEALFHVTEWKEFRM
Consensus       ...  . ..   . ..**.. .  .......     .  ..    ..*..... .. *

BP1629     398  MGAEAIRKFGKAEHILYDLKYVLAPEQADLRL--------------   429
BP3150     393  MGAEAIRKFGKAEHILYDLKYVLAPEQADLRL--------------   424
PGN_0613   407  IDLEKS---STANTIIYDVKGILPQGKKYHKL--------------   435
PA WbpA    410  ELIKAEAKLVVDSRGKYRSPAAHIIKA-------------------   436
PGN_1243   477  PDWSALSQAMAASLVIDGRNVYELPADSDFTLLNIGNSAIESASSK   522
Consensus       .  ... .   . ....... .. .   . ..
```

Supplemental Figure 3

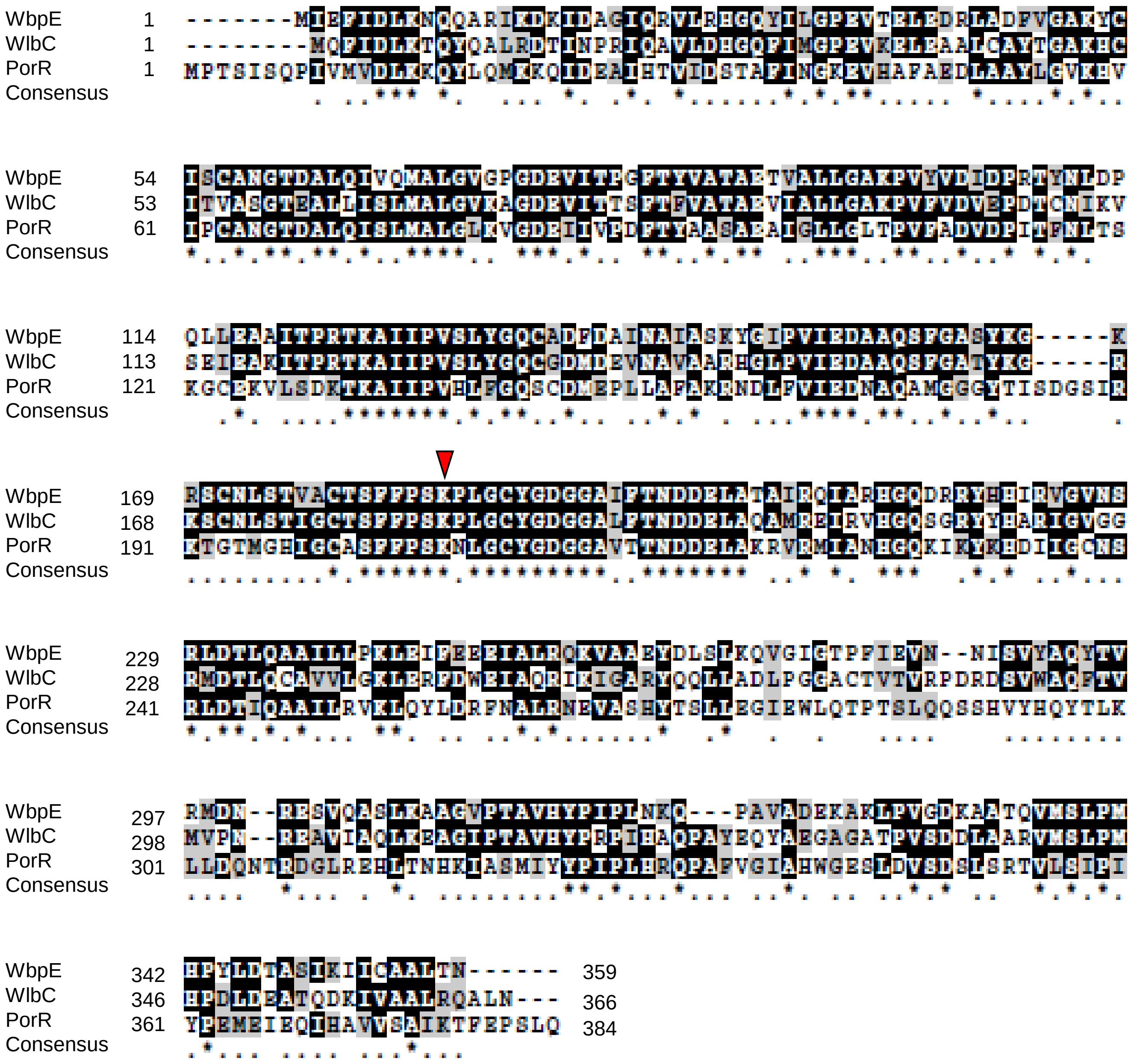

Supplemental Figure 4

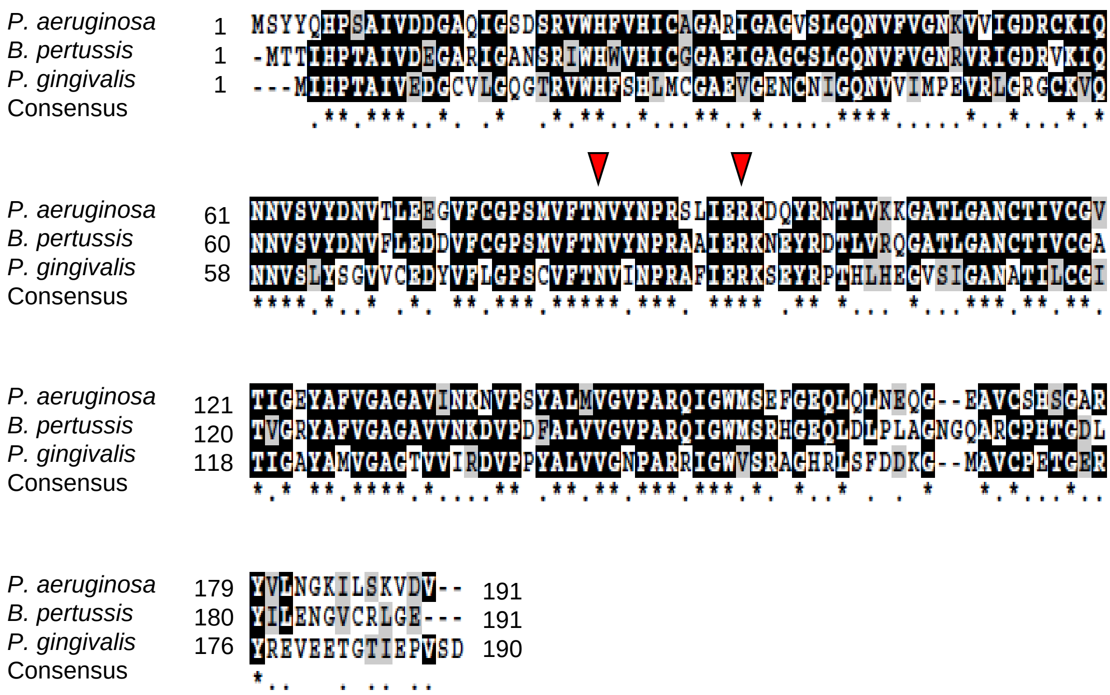

Supplemental Tabe 1. Bacterial strains used in this study.

P. gingivalis strain	Description	Source or reference
33277	Wild type	ATCC
HG66	Naturally non-pigmented strain	Gift from J. Potempa[a]
HG66/pWbpB+	HG66/pTCB-wbpB	This study
KDP220	*wbpB*::Em^r	This study
KDP221	*porR*:Tc^r	This study
KDP222	PGN_0002::Em^r	This study
KDP223	KDP222/pTCB-PGN_0002	This study
KDP224	KDP222/pTCB-P. a. wbpD	This study
Pugd1	*ugdA*::Em^r	Sato et al. 2009
W83	Wild type	Gift from M. J. Duncan[b]

E. coli strain	Description	Source or reference
S17-1	RP-4-2-Tc::Mu *aph*::Tn7 *recA*, Sm^r	Simon et al. 1983
XL-1 Blue	General purpose host strain for cloning	Stratagene

P. aeruginosa strain	Description	Source or reference
PAO1	Wild-type	Gift from J. S. Lam[c]

a Department of Molecular Genetics, The Forsyth Institute, Boston, MA.
b Faculty of Biochemistry Biophysics and Biotechnology, Jagiellonian University, Krakow, Poland.
c Department of Molecular and Cellular Biology, University of Guelph, Ontario, Canada.

Supplemental Table 2. Plasmids used in this study.

Plasmid	Description	Source or reference
pBluescript II SK(-)	Ap^r, cloning vector	Stratagene
pBSSK-cat pro-rgpBter	Ap^r, pBSSK-cat pro-rgpBter	This study
pBSSK-cat pro-wbpB-rgpBter	Ap^r, pBSSK-cat pro-wbpB-rgpBter	This study
pBSSK-cat pro-PGN_0002-rgpBter	Ap^r, pBSSK-cat pro-PGN_0002-rgpBter	This study
pBSSK-cat pro-P. a. wbpD-rgpBter	Ap^r, pBSSK-cat pro-P. a. wbpD-rgpBter	This study
pCR4	Ap^r Km^r, PCR Topo cloning vector	Invitrogen
pCR4-cat pro	Ap^r Km^r, pCR4-cat pro	This study
pCR4-porRup	Ap^r Km^r, pCR4-porRup	This study
pCR4-porRdw1	Ap^r Km^r, pCR4-porRdw1	This study
pCR4-porRdw2	Ap^r Km^r, pCR4-porRdw2 (NotI self-ligated of pCR4-porRdw1)	This study
pCR4-porRupdw2	Ap^r Km^r, pCR4-porRup-porRdw2	This study
pGEM-T Easy	Ap^r, PCR cloning vector	Promega
pKD375	Ap^r, Tc^r, pUC19-*tetQ*	Shi et al. 1999
pKD740	Ap^r Em^r, pGEM-T Easy-hbp35up-*ermF*-hbp35dw	Shoji et al. 2010
pKD854	Ap^r, pBSSK-sKGP-rgpBter	Sato et al. 2005
pKD892	Ap^r Em^r, pGEM-T Easy-PGN_0168up-*ermF*-PGN_0168dw	This study
pKD893	Ap^r Em^r, pGEM-T Easy-PGN_0002up-*ermF*-PGN_0002dw	This study
pKD894	Ap^r Km^r Tc^r, pCR4-PGN_1236up-*tetQ*-PGN_1236dw	This study
pKD955	Ap^r, pBSSK-p-porK+-T	Sato et al. 2010
pTCB	Ap^r Tc^r, shuttle vector plasmid	Nagano et al. 2007

pTCB-*wbpB*	Ap^r Tc^r, pTCB-PGN_0168	This study
pTCB-PGN_0002	Apr Tc^r, pTCB-PGN_0002	This study
pTCB-*P. a. wbpD*	Ap^r Tc^r, pTCB-wbpD from *P. aeruginoza* PAO1	This study
pUC118	Ap^r, PCR cloning vector	Takara
pUC118-*wbpB*	Ap^r, pUC118-PGN_0168	This study
pUC118-PGN_0002	Ap^r, pUC118-PGN_0002	This study
pUC118-*P.a. wbpD*	Ap^r, pUC118-*wbpD* from *P. aeruginoza* PAO1	This study

Supplemental Table 3. Primers used in this study.

Primer name	Nucleotide sequence (5'-3')	underline	double underline
p6-34-F-KpnI	GGTACCTTCGTCGTCAATCAGCATCCCAG	KpnI	
p6-34-R-SalI	GTCGACTGTTTTGTCTCTTATTTAAGTTA	SalI	
PGN_0002upFw	GCATGCTCCAGCAGCGTACGCTTCAAAGC	SphI	
PGN_0002upRev	GGATCCAAAGACCTGCTATCAATTGGCGA	BamHI	
PGN_0002dwFw	CTGCAGTCTACACCAAATAGCGGCATATC	PstI	
PGN_0002dwRev	GAGCTCCGATTACATACTCGGTTACAACC	SacI	
PGN_0168upFw	GCATGCATGCACAACTTTGCCATCATCGG	SphI	
PGN_0168upRev	GGATCCCTCGCATATCACATCCGCTCCAA	BamHI	
PGN_0168dwFw	CTGCAGAAAGGAATTTCTATAATCTTATC	PstI	
PGN_0168dwRev	GAGCTCCGCTGTGCACCGATGTCGGACAC	SacI	
PGN_1236upFw	GGTACCGTGAACGATAGCGAAGGGAAAGCA	KpnI	
PGN_1236upRev	GGATCCAGGCGTGTTGTAGGGATTTCTGGT	BamHI	
PGN_1236dwFw	GGATCCCAAAAGAGGAGGGAGGCTCGTCTA	BamHI	
PGN_1236dwRev	GCGGCCGCCAGATTATTGCCCAGACCTTGA	NotI	
PGN_0168compFw	GTCGACATGCACAACTTTGCCATCATCGGT	SalI	Start codon
PGN_0168compRev	TCTAGATTAGCGTCTCCATCCGAAAGGATG	XbaI	Stop codon
PGN_0002compFw	GTCGACATGATCCATCCCACAGCGATAGTA	SalI	Start codon
PGN_0002compRev	TCTAGATTAGTCCGATACCGGTTCTATTGT	XbaI	Stop codon
*P.a. wbpD*compFw	GTCGACATGAGTTATTATCAGCACCCCAGCGCGATCGTGGAC	SalI	Start codon
*P.a. wbpD*compRev	TCTAGATTACACGTCCACCTTGCTCAGGAT	XbaI	Stop codon
